# Effects of AI-assisted wushu training on mental health, cognitive performance, and cardiovascular function in university students: a quasi-experimental study

**DOI:** 10.3389/fpubh.2026.1932711

**Published:** 2026-09-21

**Authors:** Jiannan Yang, Ziyin Zhang, Ziwu Li

**Affiliations:** 1 Hunan Normal University, Changsha, China; 2 Hunan University of Chinese Medicine, Changsha, China; 3 Henan University of Technology, Zhengzhou, China

**Keywords:** artificial intelligence, campus health promotion, cardiovascular function, cognitive performance, martial arts, mental health, university students

## Abstract

**Background:**

In this quasi-experimental study, we examined whether AI-assisted wushu (Chinese martial arts) routine training, delivered through camera-based immediate post-performance movement feedback, was associated with greater improvements in mental health, cognitive performance, and cardiovascular function than conventional wushu instruction among university students.

**Methods:**

A campus-based, two-group, quasi-experimental pre–post design with non-randomized allocation by course section was conducted over 6 weeks among 298 physical education undergraduates (*n* = 149 per group). Outcomes included perceived stress, anxiety, psychological well-being, and emotion regulation (mental health); Stroop, digit-span, n-back, and Trail-Making Test performance and a composite executive-function index (cognitive performance); and resting heart rate, blood pressure, heart-rate variability, vital capacity, and heart-rate recovery (cardiovascular function). Post-intervention group differences were modeled with ANCOVA adjusting for baseline scores, age, sex, and BMI; associations between movement-quality gain and outcome change were examined with Pearson correlation and covariate-adjusted bootstrapped mediation, with false discovery rate correction applied.

**Results:**

Groups did not differ significantly at baseline (all *p* > 0.07). Both groups improved on every outcome (all *p* < 0.001), and after adjustment the AI-assisted group showed somewhat larger gains, including for perceived stress (adjusted mean difference −1.20, 95% CI − 1.74 to −0.66), psychological well-being (+1.15, 95% CI 0.39 to 1.91), n-back accuracy (+2.37, 95% CI 1.59 to 3.15), the executive-function index (+0.12, 95% CI 0.07 to 0.18), resting heart rate (−1.09 bpm, 95% CI − 1.58 to −0.60), and vital capacity (+51.6 mL, 95% CI 34.8 to 68.4; all *p* < 0.03, retained after FDR correction). Movement-quality gain correlated modestly with cognitive-affective but not cardiovascular improvement (*r* = 0.17–0.22, *p* < 0.01), and partially mediated group effects on emotion regulation and executive function.

**Conclusion:**

These findings therefore support a multiple-pathway interpretation of the incremental AI-feedback effect rather than a single motor-skill mechanism. Given the non-randomized design, these associations are preliminary rather than definitive evidence of causal benefit; randomized, longer-term studies with appropriate active controls are needed to confirm mechanisms, durability, and generalizability.

## Introduction

### The campus mental health burden

University life is a period of heightened vulnerability for mental health. Meta-analytic evidence places the global pooled prevalence of depressive and anxiety symptoms among college students at approximately 33.6 and 39.0%, respectively (1), and longitudinal survey data indicate that psychological well-being can decline measurably over the course of university study rather than improve with adjustment (2). Chronic psychological stress and reduced physical activity are mutually reinforcing: stress suppresses exercise motivation, while inactivity removes one of the most consistently effective non-pharmacological buffers against anxiety and depressive symptoms (3, 4). Campus health promotion programs therefore face a dual mandate—they must be attractive and sustainable enough to counteract stress-driven disengagement, while being structured enough to deliver a genuine physiological and psychological training stimulus.

### Physical activity as a multi-domain health lever

Physical activity is among the most consistently recommended interventions for these overlapping problems. The World Health Organization’s 2020 guidelines on physical activity and sedentary behaviour recommend that adults accumulate 150–300 min of moderate-intensity (or 75–150 min of vigorous-intensity) aerobic activity per week for optimal health (5), yet the benefits of structured exercise extend well beyond cardiovascular risk reduction. Aerobic and coordinative exercise reliably improves executive function, attention, and processing speed across the lifespan, an effect attributed to exercise-induced neurotrophic and cerebrovascular adaptation (6, 7), and meta-analyses of acute and chronic exercise interventions confirm reliable, if moderate, gains in working memory, inhibitory control, and cognitive flexibility (8, 9). In parallel, exercise training measurably improves autonomic cardiovascular regulation: systematic review evidence in young and middle-aged adults shows that endurance, high-intensity, and coordinative training modalities increase resting heart-rate variability and improve blood pressure and heart-rate recovery, indices of parasympathetic (vagal) tone and reduced cardiovascular risk (10). Because wushu routine practice combines moderate-intensity aerobic exertion with complex coordinative and attentional demands, it is a plausible candidate for simultaneously engaging all three of these benefit pathways within a single campus activity.

### Wushu and other mind–body traditions as campus interventions

Among culturally embedded forms of physical activity, wushu and related mind–body traditions are particularly well positioned to meet this dual mandate. Wushu (Chinese martial arts routine practice, or taolu) is both a competitive sport and a culturally embedded physical education activity in Chinese universities, and a growing body of evidence links martial arts and related mind–body traditions to psychological benefit. Tai Chi—a related traditional Chinese practice sharing wushu’s emphasis on coordinated movement, breath regulation, and sustained attentional focus—has been shown in randomized and non-randomized trials to reduce stress, anxiety, and depressive symptoms and to improve mood (11). Wushu-specific research similarly reports that structured Wushu instruction is associated with improved mental health indicators among Chinese university students (12), and randomized evidence from martial arts programs more broadly demonstrates gains in psychological resilience (13) and executive function (14) among youth populations. A broader clinical review similarly concludes that regular martial arts practice confers measurable physical and psychological health benefits across age groups (15). These findings position wushu not merely as a competitive sport but as a candidate vehicle for campus-wide psychological, cognitive, and cardiovascular health promotion—provided that instructional quality and consistency can be assured at scale.

### AI-based movement feedback as an instructional enhancement

Realizing this potential at scale, however, depends on instructional quality, which is where AI-based movement feedback becomes relevant. A persistent constraint on realizing wushu’s full health-promoting potential in large university classes is the scarcity of individualized, immediate feedback: correct execution of a routine depends on fine-grained postural and rhythmic control that a single instructor cannot continuously monitor across dozens of students. The motor-learning literature has long established that augmented feedback—information about performance supplied from an external source—can meaningfully accelerate skill acquisition when it is timely, specific, and appropriately dosed (16, 17). Recent advances in deep-learning-based human pose estimation now make it possible to deliver this kind of feedback automatically: computer-vision systems can track joint trajectories in real time and translate them into quantitative information about movement accuracy, stability, and timing that can be returned to the learner as immediate corrective feedback (18). Purpose-built systems for sports posture correction using such architectures have demonstrated high positional accuracy and robustness to occlusion and rapid movement in applied athletic settings (19). Importantly, the potential instructional advantage considered here is not that AI replaces the human instructor or is inherently more credible than human feedback. Rather, AI can augment the same instructor-led curriculum by providing individualized, visually explicit, quantitative, and consistently available error information that is difficult to deliver continuously in large classes.

### A motivational mechanism: engagement and adherence

Beyond its direct instructional value, AI-assisted feedback may also operate through a motivational pathway. Self-determination theory holds that perceived competence—a sense of mastery and visible progress—is one of the basic psychological needs whose satisfaction predicts sustained exercise engagement and adherence (20). From this perspective, any motivational advantage should be attributed to competence-supportive characteristics of the feedback—such as specificity, immediacy, individualized error information, and visible progress—rather than to the feedback being delivered by a non-human source. High-quality instructor feedback could theoretically support the same need; the practical distinction is that an automated system can provide standardized and individualized competence-relevant information repeatedly within large-group instruction. Because perceived competence and basic psychological need satisfaction were not directly measured in the present study, self-determination theory is used as an explanatory framework rather than as a formally tested mediator model.

### The present study

Taken together, this literature suggests that AI-assisted feedback may add to a shared wushu-training effect through at least two conceptually distinct pathways. First, a motor-learning pathway may operate when more explicit error information improves movement quality and intensifies cognitive-motor error correction, with downstream benefits for selected cognitive-affective outcomes. Second, an SDT-informed behavioral pathway may operate when competence-supportive feedback characteristics promote engagement and adherence, thereby increasing realized training exposure; however, the present study assesses engagement and adherence as process indicators and does not directly measure perceived competence. Because both groups completed the same wushu curriculum, including its coordinative, attentional, breathing-related, and aerobic demands, these shared components are expected to account for substantial pre-post benefit in both groups. The present study therefore focuses on the incremental association of AI-augmented feedback within wushu practice, rather than on the effect of wushu relative to a non-wushu activity.

### Research questions

*RQ1*: Does AI-assisted wushu training improve university students’ mental health (perceived stress, anxiety, psychological well-being, emotion regulation) more than conventional training?

*RQ2*: Does AI-assisted wushu training improve cognitive performance (attention, working memory, executive function, and processing speed) more than conventional training?

*RQ3*: Does AI-assisted wushu training improve cardiovascular function (resting heart rate, blood pressure, heart-rate variability, vital capacity, heart-rate recovery) more than conventional training?

*RQ4*: Is AI-assessed improvement in movement quality associated with improvement in mental health, cognitive, and cardiovascular outcomes?

*RQ5*: Does gain in AI-assessed movement quality mediate the relationship between AI-assisted training and health outcomes?

### Hypotheses

*H1*: AI-assisted training will produce significantly greater improvement in mental-health indicators than conventional training.

*H2*: AI-assisted training will produce significantly greater improvement in cognitive-performance indicators than conventional training.

*H3*: AI-assisted training will produce significantly greater improvement in cardiovascular-function indicators than conventional training.

*H4*: Gain in AI-assessed movement quality will correlate with improvement in mental-health, cognitive, and cardiovascular outcomes.

*H5*: Gain in AI-assessed movement quality will partially mediate the effect of AI-assisted training on health outcomes.

## Methods

### Conceptual framework

The AI-Enhanced Wushu Health Promotion Framework is interpreted as an AI-specific enhancement layered onto a shared wushu-training substrate. The shared substrate comprises the routine practice, coordinative demands, sustained attention, breathing-related regulation, and aerobic exercise exposure received by both groups and is expected to contribute to health improvement in both conditions. The AI-specific technology-enhancement layer uses computer-vision-based pose recognition to evaluate movement accuracy, stability, coordination, rhythm, and completeness and to return individualized visual and quantitative feedback. A behavioral/learning-adaptation layer includes movement-quality change, engagement, adherence, and exercise self-efficacy; the competence-supportive interpretation of this layer is theoretically informed by self-determination theory but was not directly measured through perceived-competence or basic-need-satisfaction scales. The health-outcome layer represents cumulative attentional, autonomic, and affective adaptation expressed as changes in mental health, cognitive performance, and cardiovascular function (Figure 1).

**Figure 1:**
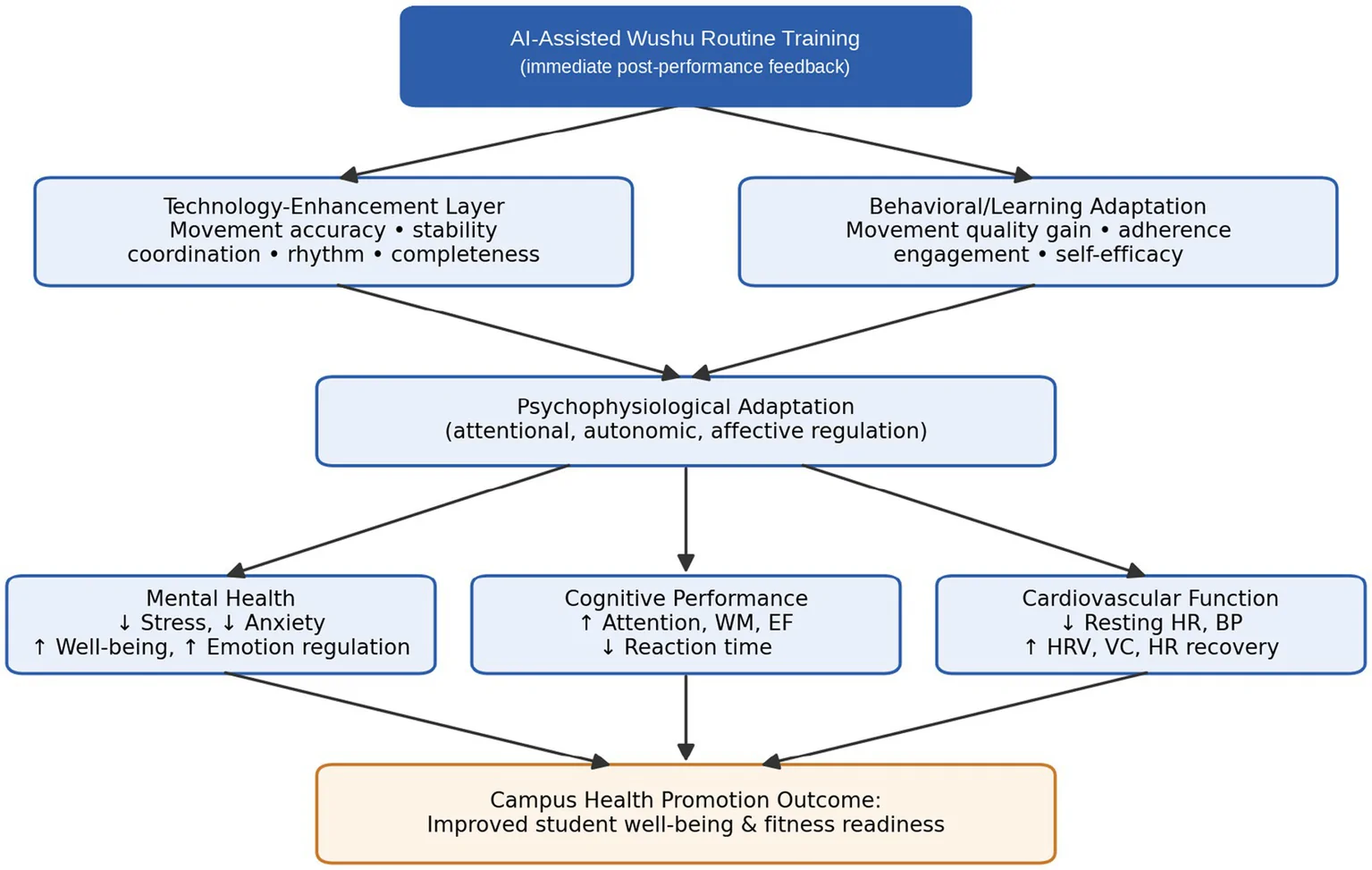
Conceptual framework linking AI-assisted movement feedback to campus health outcomes.

### Design

This was a campus-based, two-group, quasi-experimental pre–post intervention study conducted over a 6-week training program. Group assignment followed existing wushu course sections (non-randomized, convenience allocation) rather than individual randomization; as a result, between-group comparisons in this study should be interpreted as associational rather than strictly causal, and unmeasured section-level differences (e.g., instructor, scheduling, or self-selection effects) cannot be ruled out even where measured baseline characteristics are well matched (see Limitations). All outcomes were assessed at baseline (pre-intervention) and again at the end of the training program (post-intervention).

### Participants

Participants were undergraduate students enrolled in wushu courses within the Physical Education program at Hunan Normal University. Of 300 students initially recruited, 298 provided complete pre and post-intervention data and were retained for analysis (attrition = 0.7%). The analytic sample comprised 149 students in the AI-assisted wushu group and 149 in the conventional wushu group. Mean age was 20.3 ± 1.2 years (range 18–23), mean BMI was 21.8 ± 2.2 kg/m^2^, and mean prior wushu experience was 2.5 ± 2.0 years; 160 participants (53.7%) were female and 138 (46.3%) male. As shown in Table 1, the two groups did not differ significantly on any baseline demographic or outcome measure (all *p* > 0.07), supporting between-group comparability prior to intervention. Ethical approval for the study and the procedures used to obtain written informed consent from all participants prior to enrollment are described in the Ethics Statement.

**Table 1:** Baseline demographic and outcome comparability between groups.

Variable	AI-wushu (*n* = 149)	Conventional (*n* = 149)	*t*/*χ*2	*p*
Age (years)	20.31 ± 1.18	20.30 ± 1.17	0.09	0.929
BMI (kg/m^2)	21.68 ± 2.23	21.87 ± 2.21	−0.74	0.458
Wushu experience (years)	2.63 ± 2.07	2.46 ± 1.92	0.71	0.480
Perceived stress (PSS)	18.68 ± 5.22	19.36 ± 4.73	−1.18	0.240
Anxiety (GAD-7)	7.27 ± 2.88	7.32 ± 3.22	−0.14	0.890
Psychological well-being	61.66 ± 7.64	61.03 ± 8.44	0.68	0.500
Emotion regulation	38.13 ± 5.06	37.32 ± 4.63	1.45	0.148
Stroop RT (ms)	689.44 ± 71.72	691.80 ± 67.97	−0.29	0.771
Stroop accuracy (%)	86.72 ± 4.81	86.15 ± 5.42	0.95	0.342
Digit span	7.13 ± 1.29	7.17 ± 1.26	−0.26	0.792
N-back accuracy (%)	74.78 ± 8.49	75.91 ± 8.70	−1.13	0.260
TMT-B (s)	61.19 ± 10.54	62.12 ± 10.53	−0.76	0.448
Executive function (Z)	−0.01 ± 0.72	−0.05 ± 0.70	0.42	0.675
Resting HR (bpm)	76.35 ± 6.44	75.02 ± 6.57	1.76	0.079
SBP (mmHg)	116.12 ± 8.78	117.53 ± 7.80	−1.46	0.144
DBP (mmHg)	75.79 ± 5.15	75.28 ± 5.50	0.83	0.406
HRV RMSSD (ms)	37.53 ± 10.03	36.62 ± 10.33	0.76	0.446
Vital capacity (ml)	3743.45 ± 587.81	3624.12 ± 632.92	1.69	0.093
HR recovery 1-min (bpm)	26.16 ± 6.85	26.05 ± 5.38	0.16	0.877
AI movement quality score	71.35 ± 7.53	70.14 ± 7.51	1.39	0.166
Sex, female/male, *n*	81/68	79/70	*χ*^2^ = 0.01	0.907

### Intervention

Both groups completed a structured, 6-week wushu routine training program with equivalent frequency, session duration, routine content, and overall session structure: warm-up (10–15 min), fundamental technique practice (15–20 min), routine (taolu) instruction and combination practice (25–35 min), and cool-down (5–10 min). Training was delivered 2–3 times per week for 60–90 min per session (approximately 12–18 sessions in total). The principal experimental contrast concerned the modality and structure of feedback during a corresponding 10–15-min feedback/practice segment. In the AI-assisted group, the WushuPose-AI system provided individualized visual information about movement deviations, five dimension-specific scores (movement accuracy, stability, coordination, rhythm consistency, and completeness), a composite movement-quality score, and corresponding corrective suggestions. In the conventional group, the same period was supervised by the instructor, who provided verbal corrections based on usual wushu teaching practice; students did not receive automated visual feedback or algorithm-generated movement-quality scores. Thus, the comparison was not between feedback and no feedback, nor between an AI teacher and a human teacher, but between AI-augmented, quantitative and visually explicit feedback and conventional instructor-delivered verbal correction within the same instructor-led wushu curriculum.

### AI-assisted feedback system

The AI-assisted feedback system used a custom camera-based human pose-estimation pipeline (WushuPose-AI v1.2, built on a MediaPipe BlazePose full-body landmark model tracking 33 keypoints at 30 frames per second) to extract key joint landmarks during wushu routine practice. A single RGB camera (1080p resolution, 30 fps) was mounted 3–4 m in front of, and slightly elevated above, each practice area so that the student’s full body remained in frame throughout the routine. Although training sessions were conducted in a group-class format, AI-assisted evaluation was performed individually; only one participant was positioned within the designated camera capture area at a time. The system generated five sub-scores (0–100 scale) for movement accuracy, postural stability, inter-segmental coordination, rhythm consistency, and routine completeness, each derived from frame-by-frame comparison of the student’s tracked joint-angle trajectories against a reference expert-performed routine template using dynamic time warping (DTW) alignment. For each component, movement deviations relative to the reference expert template were quantified after temporal alignment and normalized to a 0–100 similarity-based scale, with smaller deviations corresponding to higher scores. These sub-scores were combined into a composite movement-quality score calculated as their unweighted mean (Composite = [Accuracy + Stability + Coordination + Rhythm + Completeness]/5; range 0–100). The five component scores and their equal weighting were specified before the instructor-validation procedure. Instructor ratings were used only to evaluate concurrent validity and were not used to train, weight, or recalibrate the automated scoring system. During the AI-assisted feedback segment, each student completed one full routine in turn. Immediately after completion, the system displayed the five dimension-specific scores and the composite movement-quality score on a computer screen, together with visual indications of detected movement deviations and corresponding corrective suggestions. Students then used this immediate post-performance feedback to guide the subsequent practice attempt. Prior to the study, concurrent validity of the automated scoring was established by comparing system-generated composite scores with independent ratings from two certified wushu instructors (blinded to condition) on a subsample of 40 practice videos; system and expert-averaged scores were strongly correlated (Pearson *r* = 0.86, *p* < 0.001), supporting the validity of the automated scores for both feedback and research purposes. For research purposes, the same system was used to score participants in both groups, but feedback was only returned to the AI-assisted group.

### Measures

#### Assessment timing

Baseline assessments were completed before the start of the 6-week intervention. Post-intervention assessments were conducted 24–48 h after the final training session rather than immediately after exercise, to minimize the influence of acute exercise responses. Mental-health, cognitive, and physiological assessments were conducted under comparable conditions at the two assessment time points.

#### Mental health

Perceived stress was assessed with the Perceived Stress Scale (PSS; (21)), anxiety with the 7-item Generalized Anxiety Disorder scale (GAD-7; (22)), and emotion regulation (ER) with a validated emotion-regulation self-report measure (23, 24), with higher PSS and GAD scores reflecting greater distress and higher psychological well-being and ER scores reflecting better functioning. Psychological well-being (PWB) was measured with the 18-item short-form Ryff Scales of Psychological Well-Being (25), comprising six three-item subscales—autonomy, environmental mastery, personal growth, positive relations with others, purpose in life, and self-acceptance—each rated on a 6-point Likert scale (1 = strongly disagree to 6 = strongly agree); subscale scores were summed to yield a total score ranging from 18 to 108, with higher scores indicating greater psychological well-being.

#### Cognitive performance

Cognitive performance was assessed with a battery of objective tasks: a computerized Stroop task (reaction time and accuracy; (26)), a digit-span task (working memory span), an n-back task (working-memory accuracy; (27)), and the Trail-Making Test Part B (TMT-B, cognitive flexibility/set-shifting, seconds to complete; (28)). A composite executive-function index (z-score) was derived from these measures, with higher values indicating better executive function.

#### Cardiovascular function

Resting heart rate and heart-rate variability were recorded using a Polar H10 heart-rate sensor (Polar Electro Oy, Kempele, Finland), with heart-rate variability expressed as the root mean square of successive RR-interval differences (RMSSD) (30). Systolic and diastolic blood pressure were measured using an automated sphygmomanometer (HEM-907, Omron Healthcare Co., Ltd., Kyoto, Japan). Vital capacity was assessed using a portable spirometer (Spirobank II, MIR—Medical International Research, Rome, Italy). One-minute heart-rate recovery (HRR1) was assessed following a standardized 3-min step test. Heart rate was recorded using the Polar H10 sensor at the end of the test and again after 1 min of recovery, and HRR1 was calculated as the difference between end-exercise heart rate and heart rate at 1 min of recovery.

#### Mechanism/process variables

AI-assessed movement quality (0–100 composite score) was recorded at baseline and post-intervention for all participants (the conventional group’s routines were also scored by the same automated system for research purposes, though without feedback being returned to students). Training adherence was assessed objectively using instructor-recorded attendance. At each scheduled training session, the instructor conducted a roll call and recorded whether each participant was present or absent; individual adherence was calculated as the number of sessions attended divided by the number of scheduled sessions, multiplied by 100. Adherence was calculated separately for each participant relative to the number of sessions scheduled for that participant’s course section; the reported group values therefore represent the mean of individual adherence percentages rather than a pooled proportion of all student-session observations. Engagement was recorded using a 1–5 self-report scale, and exercise self-efficacy was also assessed during the intervention period as a process indicator. Perceived trust in, credibility of, or preference for AI-generated versus instructor-delivered feedback was not measured, and perceived competence/basic psychological need satisfaction was not directly assessed.

#### Statistical analysis

Given the non-randomized design, covariate adjustment (baseline score, age, sex, and BMI) was used to reduce, but cannot fully eliminate, the influence of measured and unmeasured baseline group differences on post-intervention comparisons. Baseline comparability was tested with independent-samples *t*-tests (continuous variables) and chi-square tests (categorical variables). Within-group pre–post change was tested with paired-samples *t*-tests, and within-group effect sizes were expressed as Cohen’s d for paired differences. Between-group differences in post-intervention outcomes were tested using analysis of covariance (ANCOVA), regressing each post-test score on group, the corresponding baseline score, age, sex, and BMI; the group coefficient represents the baseline and covariate-adjusted mean difference between the AI-assisted and conventional groups, and partial η^2^ was calculated as the effect-size index. Associations between change in AI-assessed movement quality and change in each outcome were examined with Pearson correlations across the full sample. Mediation of the group effect on outcomes through change in movement quality was tested using the product-of-coefficients approach (29), implemented as covariate-adjusted linear regression with 1,000-replicate non-parametric bootstrap confidence intervals for the indirect (a × b) effect. Because 16 primary health outcomes and 16 exploratory movement-quality correlations were each tested as a family, we additionally applied the Benjamini–Hochberg false discovery rate (FDR) procedure within each family (*q* = 0.05); FDR-adjusted *p*-values are reported alongside nominal *p*-values in Tables 2, 3. All analyses were conducted in Python (pandas, SciPy, statsmodels); two-tailed *α* = 0.05 was used for nominal tests throughout. Between-group differences in adherence, engagement, and exercise self-efficacy were evaluated with independent-samples *t*-tests and summarized using mean differences, 95% confidence intervals, and Cohen’s d. As a sensitivity analysis, adherence and engagement were additionally modeled with linear regression adjusting for age, sex, BMI, and prior wushu experience. For adherence, distributional robustness was additionally examined using group medians and interquartile ranges (IQRs) and by repeating the between-group comparison after excluding observations outside the group-specific 1.5 × IQR bounds.

**Table 2:** Pre–post change in cognitive performance outcomes by group.

Variable	AI pre	AI post	AI d	Conv pre	Conv post	Conv d
Stroop RT (ms)	689.44 ± 71.72	666.76 ± 73.85	−1.19	691.80 ± 67.97	680.55 ± 70.04	−0.52
Stroop accuracy (%)	86.72 ± 4.81	89.89 ± 5.38	1.32	86.15 ± 5.42	88.02 ± 5.81	0.74
Digit span	7.13 ± 1.29	7.69 ± 1.42	1.07	7.17 ± 1.26	7.43 ± 1.32	0.49
N-Back accuracy (%)	74.78 ± 8.49	79.48 ± 9.07	1.30	75.91 ± 8.70	78.19 ± 8.86	0.72
TMT-B (s)	61.19 ± 10.54	56.37 ± 10.98	−1.10	62.12 ± 10.53	59.12 ± 11.01	−0.69
Executive function (Z)	−0.01 ± 0.72	0.25 ± 0.74	1.15	−0.05 ± 0.70	0.09 ± 0.75	0.59

**Table 3:** Pre–post change in cardiovascular function outcomes by group.

Variable	AI pre	AI post	AI d	Conv pre	Conv post	Conv d
Resting HR (bpm)	76.35 ± 6.44	74.06 ± 6.98	−1.07	75.02 ± 6.57	73.78 ± 7.21	−0.58
SBP (mmHg)	116.12 ± 8.78	113.75 ± 9.03	−0.74	117.53 ± 7.80	116.08 ± 8.36	−0.44
DBP (mmHg)	75.79 ± 5.15	74.16 ± 5.62	−0.70	75.28 ± 5.50	74.61 ± 5.82	−0.29
HRV RMSSD (ms)	37.53 ± 10.03	41.22 ± 11.66	0.76	36.62 ± 10.33	38.98 ± 11.70	0.44
Vital capacity (ml)	3743.45 ± 587.81	3842.77 ± 580.48	1.26	3624.12 ± 632.92	3674.93 ± 637.01	0.76
HR recovery 1-min (bpm)	26.16 ± 6.85	28.18 ± 7.39	0.78	26.05 ± 5.38	27.25 ± 5.95	0.48

A post-hoc sensitivity check confirmed that the study was adequately powered for the effects observed: with *n* = 149 per group, two-tailed *α* = 0.05, the design had > 99% power to detect the smallest significant between-group ANCOVA effect reported here (partial η^2^ = 0.017, equivalent to *f*^2^ ≈ 0.017) and ≥ 80% power to detect effects as small as *f*^2^ ≈ 0.02–0.03. No prospective *a priori* power calculation was performed before data collection, which is noted as a limitation. Internal-consistency reliability (Cronbach’s *α*) for the multi-item self-report scales (PSS, GAD-7, PWB, ER) could not be computed from the dataset available for this analysis, which contained only total scale scores rather than item-level responses; this is likewise noted as a limitation, and item-level reliability statistics should be reported in any subsequent submission.

## Results

### Sample characteristics and training-process fidelity

Training adherence, based on instructor-recorded roll call rather than participant self-report, was significantly higher in the AI-assisted group than in the conventional group [89.11 ± 4.95% vs. 85.44 ± 5.53%; mean difference = 3.67 percentage points, 95% CI (2.47, 4.86), *t*(296) = 6.03, *p* < 0.001, Cohen’s *d* = 0.70]. The difference remained essentially unchanged after adjustment for age, sex, BMI, and prior wushu experience (adjusted difference = 3.68 percentage points, 95% CI [2.47, 4.89], *p* < 0.001). Median adherence was 89.2% (IQR 85.9–92.1%) in the AI-assisted group and 85.9% (IQR 82.0–88.9%) in the conventional group. Three observations were identified as outliers by the group-specific 1.5 × IQR criterion (one AI-assisted and two conventional). Excluding these observations yielded a virtually unchanged between-group difference of 3.56 percentage points (*p* < 0.001, Cohen’s d = 0.70), indicating that the adherence difference was not driven by extreme observations. Self-reported engagement was also higher in the AI-assisted group (4.10 ± 0.42 vs. 3.83 ± 0.49; mean difference = 0.27, 95% CI [0.17, 0.38], *t*(296) = 5.13, *p* < 0.001, Cohen’s d = 0.59), and this difference remained significant after covariate adjustment (adjusted difference = 0.27, 95% CI [0.17, 0.38], *p* < 0.001). Exercise self-efficacy did not differ significantly between groups (32.10 ± 4.00 vs. 31.47 ± 3.68; *p* = 0.156). Both groups improved their AI-assessed movement-quality scores substantially from pre to post-intervention (AI-assisted: +7.63 points, *d* = 2.49; conventional: +4.91 points, d = 1.53; both *p* < 0.001), and the AI-assisted group’s gain remained larger after covariate adjustment (adjusted difference = 2.69, 95% CI 1.98 to 3.41, *p* < 0.001; Table 4).

**Table 4:** Training-process fidelity and AI-assessed movement quality by group.

Process indicator	AI-wushu (*n* = 149)	Conventional (*n* = 149)	Group comparison
Training adherence, % (roll-call recorded)	89.1 ± 5.0	85.4 ± 5.5	*t*(296) = 6.03, *p* < 0.001
Engagement score (1–5)	4.10 ± 0.42	3.83 ± 0.49	*t*(296) = 5.13, *p* < 0.001
Exercise self-efficacy	32.10 ± 4.00	31.47 ± 3.68	*t*(296) = 1.42, *p* = 0.156
AI movement quality—pre	71.35 ± 7.53	70.14 ± 7.51	*t*(296) = 1.39, *p* = 0.166
AI movement quality—post	78.97 ± 8.15	75.05 ± 8.11	ANCOVA-adj. Δ = 2.69, *p* < 0.001
AI movement quality—Δ (within-group)	+7.63 (d = 2.49)	+4.91 (d = 1.53)	both *p* < 0.001

### Mental health outcomes

Both groups showed significant pre-to-post improvement across all four mental-health indicators (all *p* < 0.001; Table 5), but improvements were consistently larger in the AI-assisted group (e.g., PSS decreased by 3.11 points, d = −1.36, vs. 1.90 points, d = −0.80 in the conventional group). After adjustment for baseline scores and covariates, the AI-assisted group showed lower adjusted post-intervention perceived stress (adjusted mean difference −1.20, 95% CI − 1.74 to −0.66, *p* < 0.001), lower anxiety (−0.40, 95% CI − 0.73 to −0.07, *p* = 0.018), higher psychological well-being (+1.15, 95% CI 0.39 to 1.91, *p* = 0.003), and higher emotion regulation (+1.01, 95% CI 0.56 to 1.47, *p* < 0.001) than the conventional group (Table 6), consistent with H1.

**Table 5:** Pre–post change in mental health outcomes by group.

Variable	AI pre	AI post	AI d	Conv pre	Conv post	Conv d
Perceived stress (PSS)	18.68 ± 5.22	15.57 ± 5.68	−1.36	19.36 ± 4.73	17.46 ± 5.39	−0.80
Anxiety (GAD-7)	7.27 ± 2.88	6.09 ± 3.14	−0.83	7.32 ± 3.22	6.53 ± 3.36	−0.53
Psychological well-being	61.66 ± 7.64	64.67 ± 8.42	0.89	61.03 ± 8.44	62.89 ± 9.23	0.56
Emotion regulation	38.13 ± 5.06	40.39 ± 5.25	1.24	37.32 ± 4.63	38.57 ± 5.08	0.59

**Table 6:** ANCOVA-adjusted between-group differences—mental health.

Outcome	Adj. mean diff (AI—Conv)	95% CI	*p* (raw)	*p* (FDR)	Partial η2
Perceived stress (PSS)	−1.20	[−1.74, −0.66]	<0.001	<0.001	0.062
Anxiety (GAD-7)	−0.40	[−0.73, −0.07]	0.018	0.020	0.019
Psychological well-being	1.15	[0.39, 1.91]	0.003	0.004	0.030
Emotion regulation	1.01	[0.56, 1.47]	<0.001	<0.001	0.061

### Cognitive performance outcomes

All cognitive measures improved significantly within both groups (all *p* < 0.001; Table 2). The AI-assisted group showed greater adjusted post-intervention performance than the conventional group across the cognitive battery, including faster Stroop reaction time (−11.6 ms, 95% CI − 16.2 to −6.9, *p* < 0.001), higher Stroop accuracy (+1.29%, *p* < 0.001), greater digit span (+0.30, *p* < 0.001), higher n-back accuracy (+2.37%, 95% CI 1.59 to 3.15, *p* < 0.001), faster TMT-B completion (−1.81 s, *p* < 0.001), and a higher composite executive-function index (+0.12 SD, 95% CI 0.07 to 0.18, *p* < 0.001) (Table 7), consistent with H2.

**Table 7:** ANCOVA-adjusted between-group differences—cognitive performance.

Outcome	Adj. mean diff (AI—Conv)	95% CI	*p* (raw)	*p* (FDR)	Partial η2
Stroop RT (ms)	−11.57	[−16.21, −6.94]	<0.001	<0.001	0.076
Stroop accuracy (%)	1.29	[0.73, 1.86]	<0.001	<0.001	0.065
Digit span	0.30	[0.18, 0.42]	<0.001	<0.001	0.077
N-back accuracy (%)	2.37	[1.59, 3.15]	<0.001	<0.001	0.110
TMT-B (s)	−1.81	[−2.80, −0.81]	<0.001	<0.001	0.042
Executive function (Z)	0.12	[0.07, 0.18]	<0.001	<0.001	0.070

### Cardiovascular function outcomes

Cardiovascular indicators improved significantly within both groups (all *p* < 0.001; Table 3). Adjusted between-group comparisons favored the AI-assisted group for resting heart rate (−1.09 bpm, 95% CI − 1.58 to −0.60, *p* < 0.001), systolic blood pressure (−0.92 mmHg, *p* = 0.015), diastolic blood pressure (−0.96 mmHg, *p* < 0.001), HRV RMSSD (+1.32 ms, *p* = 0.027), vital capacity (+51.6 mL, 95% CI 34.8 to 68.4, *p* < 0.001), and one-minute heart-rate recovery (+0.81 bpm, *p* = 0.007) (Table 8). Effect sizes for cardiovascular differences (partial η^2^ = 0.017–0.111) were generally smaller than those for cognitive outcomes, but all were statistically significant, supporting H3.

**Table 8:** ANCOVA-adjusted between-group differences—cardiovascular function.

Outcome	Adj. mean diff (AI—Conv)	95% CI	*p* (raw)	*p* (FDR)	Partial η2
Resting HR (bpm)	−1.09	[−1.58, −0.60]	<0.001	<0.001	0.061
SBP (mmHg)	−0.92	[−1.66, −0.18]	0.015	0.017	0.020
DBP (mmHg)	−0.96	[−1.48, −0.43]	<0.001	<0.001	0.042
HRV RMSSD (ms)	1.32	[0.15, 2.49]	0.027	0.027	0.017
Vital capacity (ml)	51.60	[34.80, 68.39]	<0.001	<0.001	0.111
HR recovery 1-min (bpm)	0.81	[0.22, 1.39]	0.007	0.009	0.025

### Adjusted between-group effects: summary

Table 4 summarizes ANCOVA-adjusted between-group mean differences for all outcomes. Adjusted differences favored the AI-assisted group across all outcomes examined, with comparatively larger standardized effects observed for AI movement quality (partial η^2^ = 0.158), vital capacity (0.111), n-back accuracy (0.110), Stroop reaction time and digit span (≈ 0.076–0.077), and perceived stress and emotion regulation (≈ 0.062 each); the smallest, though still statistically significant, effects were observed for HRV RMSSD (0.017) and anxiety (0.019). All 16 primary health-outcome comparisons remained statistically significant after Benjamini–Hochberg FDR correction for multiple testing (all FDR-adjusted *p* < 0.03; Tables 6–8), suggesting the between-group differences are unlikely to reflect inflation of Type I error from testing multiple outcomes; this does not, however, establish that the differences are causally attributable to the AI-feedback component specifically (see Limitations) Table 9.

**Table 9:** ANCOVA-adjusted between-group difference—AI movement quality.

Outcome	Adj. mean diff (AI—Conv)	95% CI	*p*	Partial η2
AI movement quality	2.69	[1.98, 3.41]	<0.001	0.158

### Association between AI movement-quality gain and health outcomes

Across the full sample, change in AI-assessed movement quality (Δ AI movement quality) correlated significantly with improvement in emotion regulation (*r* = 0.216, *p* < 0.001), digit span (*r* = 0.170, *p* = 0.003), n-back accuracy (*r* = 0.182, *p* = 0.002), and the executive-function index (*r* = 0.224, *p* < 0.001), and showed a marginal trend with diastolic blood pressure (*r* = −0.108, *p* = 0.062). Movement-quality gain was not significantly associated with changes in perceived stress, anxiety, psychological well-being, Stroop performance, TMT-B, resting heart rate, systolic blood pressure, HRV, vital capacity, or heart-rate recovery (all *p* > 0.09; Table 10). These four significant associations (emotion regulation, digit span, n-back accuracy, executive-function index) survived Benjamini–Hochberg FDR correction across the 16 correlations tested (all FDR-adjusted *p* ≤ 0.013), while none of the twelve non-significant associations approached significance after correction (all FDR-adjusted *p* ≥ 0.20). H4 was therefore supported specifically, and robustly, for the cognitive-affective outcomes (emotion regulation and executive-function-related measures) but not for mental-health symptom levels or most cardiovascular indicators.

**Table 10:** Correlations between ΔAI movement quality and Δ outcomes (full sample, *n* = 298).

Outcome change (Δ)	*r*	*p* (raw)	*p* (FDR)	Sig. after FDR
Δ Emotion regulation	0.216	<0.001	0.002	Yes
Δ Digit span	0.170	0.003	0.013	Yes
Δ N-Back accuracy	0.182	0.002	0.009	Yes
Δ Executive function (Z)	0.224	<0.001	0.002	Yes
Δ Stroop RT (ms)	−0.096	0.098	0.231	No
Δ Stroop accuracy	0.058	0.314	0.369	No
Δ TMT-B (s)	0.016	0.779	0.779	No
Δ Perceived stress (PSS)	−0.073	0.209	0.359	No
Δ Anxiety (GAD-7)	−0.059	0.313	0.369	No
Δ Psychological well-being	−0.067	0.247	0.359	No
Δ Resting HR	−0.092	0.113	0.231	No
Δ SBP	−0.018	0.757	0.779	No
Δ DBP	−0.108	0.062	0.198	No
Δ HRV RMSSD	0.091	0.115	0.231	No
Δ Vital capacity	0.069	0.235	0.359	No
Δ HR recovery 1-min	0.057	0.323	0.369	No

### Mediation analysis

Bootstrapped mediation models (1,000 resamples, covariate-adjusted) tested whether gain in AI-assessed movement quality mediated the effect of group assignment (AI-assisted vs. conventional) on the two outcomes most strongly correlated with movement-quality gain in section 3.6, plus the two working-memory indicators. The indirect effect of group on emotion-regulation improvement through movement-quality gain was significant (indirect effect = 0.234, 95% bootstrap CI 0.030 to 0.433), as was the indirect effect on executive-function improvement (0.026, 95% CI 0.004 to 0.053); in both cases the direct effect of group remained significant after accounting for the mediator, indicating partial mediation. In contrast, the indirect effects for digit span (0.030, 95% CI − 0.027 to 0.085) and n-back accuracy (0.144, 95% CI − 0.163 to 0.481) were not statistically significant, indicating that movement-quality improvement did not account for these specific working-memory gains. These results provide partial support for H5 and reinforce the interpretation that movement-quality gain is a selective rather than a general mechanism across outcomes Table 11.

**Table 11:** Bootstrapped mediation: AI movement-quality gain as mediator of group effects on selected outcomes.

Outcome	Indirect effect (a × b)	95% Bootstrap CI	Significant
Δ Emotion regulation	0.234	[0.030, 0.433]	Yes
Δ Executive function (Z)	0.026	[0.004, 0.053]	Yes
Δ Digit span	0.030	[−0.027, 0.085]	No
Δ N-Back accuracy	0.144	[−0.163, 0.481]	No

## Discussion

### Summary of findings

In this quasi-experimental study of 298 university students, AI-assisted wushu routine training was associated with somewhat greater improvement than conventional instruction across all three targeted domains—mental health, cognitive performance, and cardiovascular function—despite both groups improving meaningfully from a structurally identical training program. The consistency of the direction of effect across seventeen distinct outcome measures, combined with well-matched baseline characteristics, is compatible with a contribution from the AI-feedback component, though the non-randomized design means unmeasured section-level differences cannot be fully excluded as alternative explanations (see Limitations). At the same time, effect sizes for the between-group difference (partial η^2^ typically 0.02–0.16) were considerably smaller than the very large within-group pre-post effects (Cohen’s d often > 1.0) common to both arms, indicating that wushu training itself—with or without AI feedback—is the primary driver of benefit, while AI feedback is associated with an additional, statistically significant, but comparatively modest, incremental gain. Accordingly, the between-group contrast should be interpreted as the incremental association of AI-augmented feedback within a common wushu curriculum, not as evidence that AI itself is responsible for the full health effect of wushu practice.

Both groups received corrective feedback, so the between-group difference should be interpreted as an incremental effect associated with the AI-assisted feedback format rather than feedback versus no feedback. The AI-assisted condition combined immediate post-performance delivery, individualized visual information, quantitative dimension-specific and composite scores, and corrective suggestions; this condition was associated with larger movement-quality gains and higher engagement and adherence than conventional verbal correction. Feedback latency, comparative feedback accuracy, and perceived usefulness were not directly measured, so the present study cannot determine whether AI feedback was faster, more accurate, or perceived as more useful than instructor feedback.

### Mechanisms: mental health

Wushu routine practice integrates rhythmic movement, coordinated breathing, sustained attentional focus, aerobic-coordinative exercise, and structured social participation. Because these features were common to both conditions, they provide a plausible explanation for much of the substantial pre-post improvement observed in both groups and cannot, by themselves, explain the incremental AI-versus-conventional difference. Importantly, change in movement quality was not significantly associated with change in perceived stress, anxiety, or psychological well-being. Improved technical execution should therefore not be treated as a general explanation for the broader well-being benefits. The additional psychological advantage in the AI-assisted group may instead reflect a combination of more explicit self-monitoring and a more engaging training process. The AI-assisted group showed objectively higher attendance and higher self-reported engagement; however, these process differences were not established as formal mediators of the mental-health outcomes and should therefore be interpreted as plausible complementary pathways rather than definitive causal explanations. Although SDT offers a plausible framework for interpreting the observed engagement and adherence differences, this study was not designed to test its core hypotheses. Perceived competence and psychological need satisfaction were not directly measured, and trust in the feedback source was also not assessed. Therefore, the present findings should not be cited as evidence that AI-assisted feedback supported perceived competence or validated an SDT-based mechanism. Future studies should directly measure perceived competence and psychological need satisfaction to test SDT-based predictions. Likewise, because trust in the feedback source was not assessed, the findings should not be interpreted as showing that students trusted AI-generated feedback more than instructor feedback.

The adherence difference was not driven by a small number of extreme observations. Median adherence was higher in the AI-assisted group, and excluding the three IQR-defined outliers yielded an essentially unchanged between-group effect (3.56 percentage points, *p* < 0.001, Cohen’s *d* = 0.70). Thus, the adherence result reflects a modest but broadly distributed difference in realized training exposure. However, because feedback source and format were not experimentally separated, the present data cannot identify which specific feature of the AI-assisted feedback contributed to higher attendance.

### Mechanisms: cognitive performance

Cognitive outcomes showed the clearest evidence of a movement-quality pathway: gains in AI-assessed movement quality correlated with, and partially mediated, improvement in emotion regulation and the composite executive-function index. These findings suggest that the relevant mechanism may not be movement accuracy in isolation, but the iterative cognitive-motor work required to identify errors, update an action plan, and execute corrected movements. Learning and refining a complex memorized sequence recruits working memory, executive control, and sustained attention, and visually explicit AI feedback may make this error-correction cycle more salient than verbal correction alone. At the same time, the absence of significant mediation for digit span and n-back accuracy shows that movement-quality improvement is not a universal explanation for cognitive gain. Mental effort and attentional allocation during correction were not directly measured, so the proposed cognitive-motor error-correction account remains a theoretically plausible interpretation rather than a demonstrated mechanism.

### Mechanisms: cardiovascular function

Both training conditions produced statistically significant improvements in resting heart rate, blood pressure, HRV, vital capacity, and heart-rate recovery, consistent with wushu’s moderate-intensity, whole-body aerobic and coordinative demands. These shared training characteristics, rather than technical accuracy alone, are the most direct explanation for the substantial cardiovascular improvement seen in both groups. The additional cardiovascular advantage in the AI-assisted group was comparatively modest and was not correlated with movement-quality gain, arguing against a direct physiological effect of more accurate technique. Higher objectively recorded adherence in the AI-assisted group offers one plausible alternative pathway because greater attendance increases cumulative exposure to the aerobic-coordinative training stimulus. However, adherence was not established as a formal mediator of cardiovascular change in the present analysis, so this explanation should remain tentative. Breathing patterns and attentional engagement were not separately quantified, and both groups received the same wushu curriculum; the present design therefore cannot determine the independent contribution of these mind–body components to cardiovascular or psychological change.

### Public health and practical implications

If replicated, these findings would suggest that AI-based movement feedback could serve as a low-cost, non-pharmacological addition to existing wushu curricula, potentially useful in large physical-education classes where instructor-to-student ratios limit individualized correction. The consistent, if modest, advantage across mental-health, cognitive, and cardiovascular outcomes raises the possibility that a single activity could contribute to more than one campus health-promotion priority at once, which may be attractive from a resource-allocation standpoint given the scale of untreated psychological distress documented among university students. From a practice standpoint, the pattern of results—comparatively larger adjusted differences for mental-health and cognitive outcomes than for cardiovascular indicators—suggests that AI feedback may be most usefully positioned as a complement to, rather than a substitute for, existing student-counseling and physical-education services, with instructors reasonably prioritizing deployment where cognitive-affective benefit, rather than cardiovascular conditioning, is the primary programmatic goal. However, this study did not assess implementation cost, instructor burden, technical infrastructure requirements, or feasibility outside a Physical Education curriculum at one university, so claims about scalability or broader applicability would be premature. Whether the approach generalizes to non-PE students, other institutions, or other traditional movement practices (e.g., tai chi, qigong) that share structural features with wushu is an empirical question for future research rather than a conclusion supported by the present data. The present findings suggest that AI systems intended to complement group instruction should prioritize immediate post-performance, individualized, visually explicit, quantitative feedback that can be used in the next practice attempt, while retaining instructor oversight. Future studies should experimentally isolate feedback timing, accuracy, format, and perceived usefulness before concluding which feature maximizes benefit.

## Limitations

Group assignment followed intact course sections rather than individual randomization. Although the groups were well matched on measured baseline characteristics, unmeasured confounding due to instructor effects, course-section self-selection, scheduling, or other section-level differences cannot be excluded.

The sample was drawn from Physical Education majors at a single university, who have above-average baseline fitness and prior wushu experience; generalizability to non-PE students or other institutions is uncertain.

Mental-health and engagement measures relied on self-report and were therefore vulnerable to social-desirability bias and demand characteristics. Participants were not blinded to their condition, and those in the AI-assisted group were aware that they were receiving a novel and visibly different form of feedback. Consequently, social-desirability bias and expectancy-related demand characteristics may have inflated the reported mental-health improvements in the visible AI condition. Training adherence, in contrast, was derived from instructor-recorded roll call at each scheduled session rather than participant self-report. Future studies should use blinded outcome assessors where feasible and incorporate objective stress-related measures, such as cortisol or heart-rate variability, alongside self-reported mental-health outcomes.

Perceived trust in, credibility of, and preference for AI-generated versus instructor-delivered feedback were not assessed. In addition, feedback source and feedback format co-varied across conditions: the AI-assisted condition was automated, visual, quantitative, and individualized, whereas conventional correction was instructor-delivered and verbal. The present design therefore cannot determine whether any incremental effect reflects source, format, immediacy, quantification, novelty, or a combination of these characteristics, and it should not be interpreted as evidence that students trusted AI more than human feedback.

Perceived competence and basic psychological need satisfaction were not directly measured, and exercise self-efficacy did not differ significantly between groups. Consequently, self-determination theory should be regarded as an explanatory framework for the competence-supportive characteristics of the feedback rather than as a mechanism demonstrated by a measured mediation pathway in this study.

Because both groups received wushu training, the study cannot isolate the contribution of wushu-specific mind–body components—including coordinated breathing, sustained attention, rhythmic movement, and complex coordination—from general physical-activity effects. Breathing patterns and attentional engagement were not independently quantified. A future three-arm randomized trial comparing AI-assisted wushu, conventional wushu, and an active non-wushu exercise condition matched for duration, intensity, instructor contact, and social interaction would be needed to separate the shared effect of wushu practice from the incremental effect of AI-assisted feedback.

Although objectively recorded adherence was significantly higher in the AI-assisted group with a moderate standardized difference, adherence was not formally established as a mediator of the broader health outcomes. It should therefore be interpreted as evidence of a process difference and a plausible behavioral pathway, not as a definitive explanation for all between-group effects.

The study captured only pre and post-intervention timepoints; the durability of gains after training ends, and the trajectory of change during the intervention, were not assessed.

Although the AI movement-quality scoring algorithm’s concurrent validity against expert ratings was established on a 40-video subsample prior to the study (*r* = 0.86), it was not independently re-validated against expert judges within the main analytic sample itself, and residual measurement error in this construct may have attenuated observed mediation effects.

As a quasi-experimental design, causal claims should be interpreted cautiously; randomized controlled trials are needed to confirm the incremental effect of AI feedback isolated from other differences between course sections.

Item-level responses for the self-report scales were not available for this analysis, so internal-consistency reliability (Cronbach’s α) could not be verified in the present sample; published reliability estimates for these instruments should not be assumed to transfer directly, and item-level data should be reported in the submitted version.

No prospective *a priori* power calculation was conducted before recruitment. Although the post-hoc sensitivity analysis indicated that the achieved sample size was adequate for the observed effects, future confirmatory trials should conduct and record an *a priori* power calculation prospectively.

## Conclusion

Because participants were assigned according to existing course sections rather than through randomization, unmeasured instructor, self-selection, section-level, and novelty-related effects cannot be excluded. These associations should be interpreted as promising preliminary evidence, not as definitive causal effects. Within these limitations, AI-assisted wushu routine training was associated with modestly larger improvements in mental health, cognitive performance, and cardiovascular function than conventional wushu instruction, in addition to the substantial improvements observed in both groups. Movement-quality gain was associated with improvement in emotion regulation and executive function, whereas broader mental-health and cardiovascular changes were not explained by movement-quality improvement, suggesting that multiple pathways may be involved. The higher adherence and engagement observed in the AI-assisted group represent plausible behavioral pathways but should not be interpreted as established mediators. Randomized, longer-term, multi-site studies are needed to confirm these associations, clarify the underlying mechanisms, and assess their durability and generalizability.

## Data Availability

The original contributions presented in the study are included in the article/Supplementary material, further inquiries can be directed to the corresponding author.
